# Supplementary figures and images for: Efficacy and Safety of Renal Function on Edoxaban Versus Warfarin for Atrial Fibrillation: A Systematic Review and Meta-Analysis
Source: Medicines (Basel). 2023 Jan 16;10(1):13. doi: 10.3390/medicines10010013 (PMC9861612; doi:10.3390/medicines10010013)

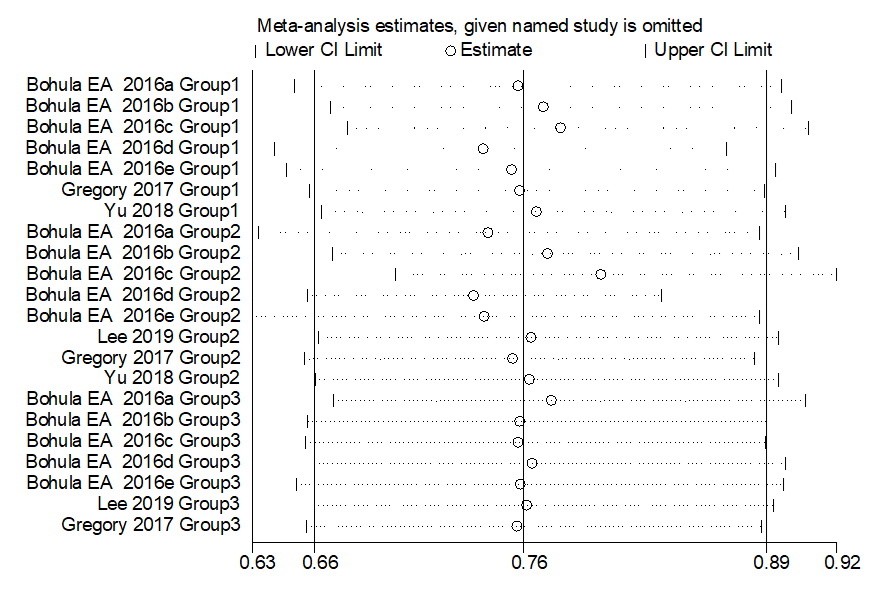

Supplement: Supplementary file 1 [file medicines-10-00013-s001.zip › Figure S1.jpg]

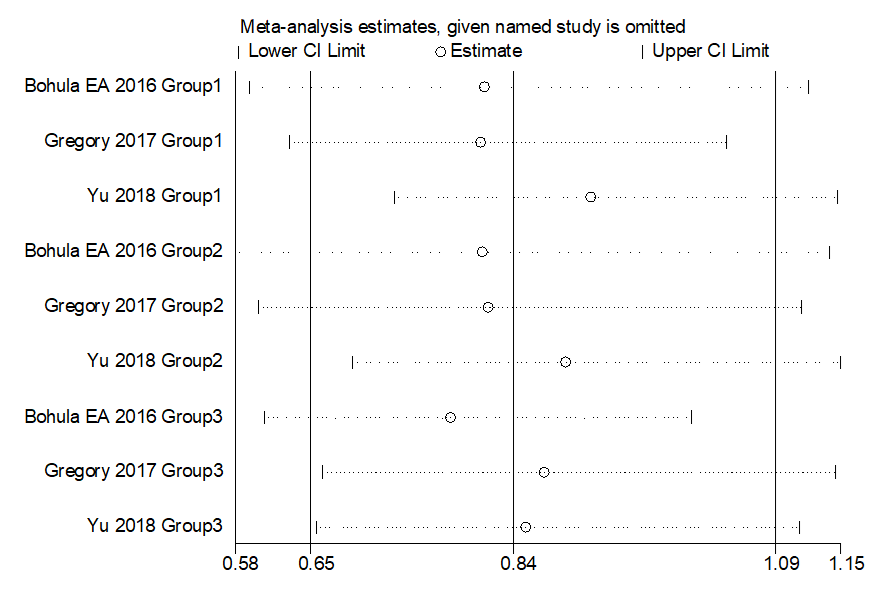

Supplement: Supplementary file 1 [file medicines-10-00013-s001.zip › Figure S2.jpg]

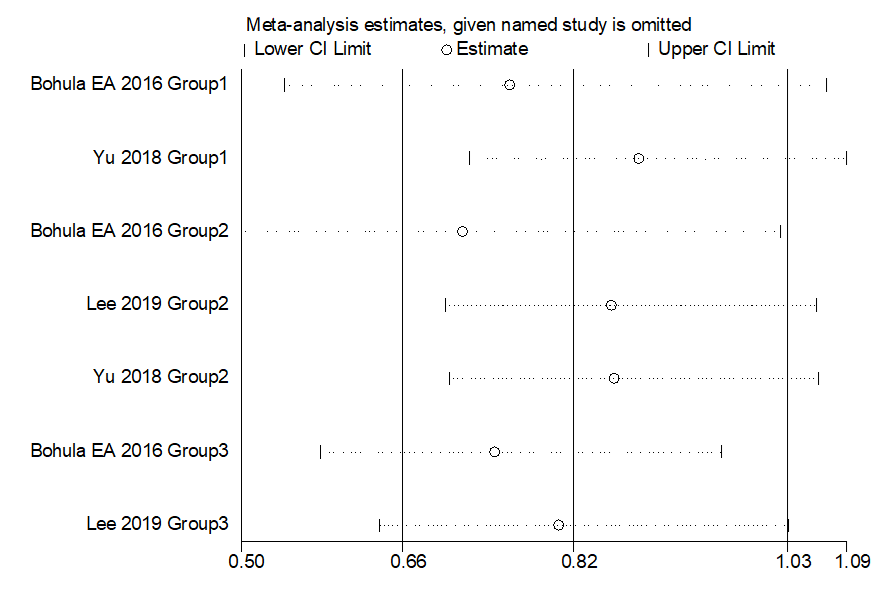

Supplement: Supplementary file 1 [file medicines-10-00013-s001.zip › Figure S3.jpg]

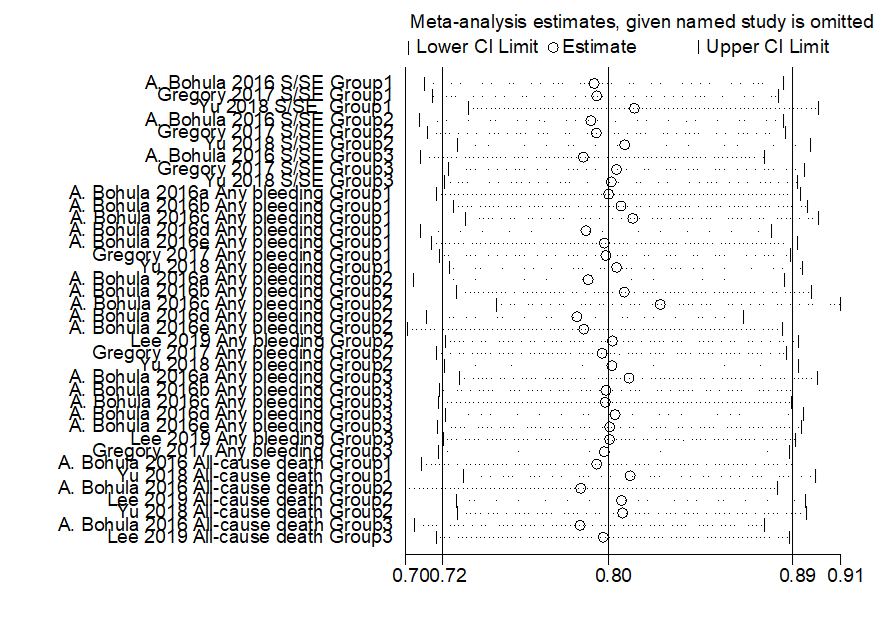

Supplement: Supplementary file 1 [file medicines-10-00013-s001.zip › Figure S4.jpg]
